# Structure, Mechanical, and Micro-Scratch Behavior of Ta-Hf-C Solid Solution Coating Deposited by Non-Reactive Magnetron Sputtering

**DOI:** 10.3390/ma15134489

**Published:** 2022-06-25

**Authors:** Zhenyu Tan, Xiang Wu, Guo Yang, Jinwei Guo, Wang Zhu

**Affiliations:** 1 Key Laboratory of Key Film Materials and Application for Equipment (Hunan Province), School of Materials Science and Engineering, Xiangtan University, Xiangtan 411105, China; zhenyutan588@163.com (Z.T.); 18374820018@163.com (X.W.); 201921001483@smail.xtu.edu.cn (G.Y.); 2 Key Laboratory of Low Dimensional Materials and Application Technology of Ministry of Education, School of Materials Science and Engineering, Xiangtan University, Xiangtan 411105, China

**Keywords:** Ta-Hf-C solid solution coating, mechanical properties, micro-scratch behavior, non-reactive magnetron sputtering

## Abstract

The TaC, HfC, and Hf-Ta-C coatings are successfully prepared by non-reactively DC magnetron sputtering. The effects of working pressure and deposition temperature on the structure and mechanical properties of Ta-Hf-C coating are analyzed. The scratch performance of the Ta-Hf-C coating deposited on 304 stainless steel and tungsten substrates are investigated. Results show the hardness and elastic modulus of Ta-Hf-C solid solution coating both increase to 37.8 ± 1.1 GPa and 435.8 ± 13.8 GPa due to the solid solution strengthening effect. Plastic deformation resistance *H*^3^/*E*^2^ of Ta-Hf-C coating can reach 0.285, which is more than twice that of binary coating. Furthermore, the scratch performance and failure mechanism show that Ta-Hf-C coating has a weaker plastic deformation resistance on soft substrate and low friction characteristic (0.01) on hard substrate, which implies that Ta-Hf-C coating is a good protective coating that can be applied to cutting tools.

## 1. Introduction

Binary carbides TaC and HfC are one of the potential candidate coating materials for high wear parts such as high temperature bearings and tools [1,2]. The attractive thing is that ternary Ta-Hf-C solid solution ceramics formed by TaC and HfC have been proven to have the higher hardness (~30 GPa) and the highest melting point temperature (>4200 K) from experimental and computational studies, which has been incorporated into the surface engineering wear-resistant material [3,4,5]. Many methods have been developed for the synthesis of Ta-Hf-C solid solution coatings, including plasma spraying, chemical vapor deposition, and physical vapor deposition [6,7]. Based on copolyfocal magnetron sputtering, Yate et al. [8] first realized the deposition of Ta-Hf-C film on stainless steel substrate. Gu et al. [9] prepared the Ta-Hf-C coating with hardness of 36 GPa and friction coefficient of 0.3 by the reactive magnetron sputtering. Noteworthily, H. Lasfargues et al. [10] reported that HfC and TaC coatings can be obtained by non-reactive sputtering with compound target. This technique is advantageous over the reactive magnetron sputtering (e.g., easier process control, fewer scattering effects, low energy consumption, and good performance of the coating). In addition, the relationship between the reaction gas flow and process parameters of traditional reactive magnetron sputtering is complex, and the hysteresis effect of process during the deposition of multicomponent system can easily lead to unstable component regions [11]. However, non-reactive magnetron sputtering of multicomponent Ta-Hf-C coatings has hardly been reported. Although non-reactive sputtering seems to show better application prospects for multicomponent coating systems, its potential has not been fully tapped. Moreover, the wear characteristics of hard coating mainly focus on material characteristics at present. However, little attention has been paid to the effect of substrate on the micro-scratch performance of the Ta-Hf-C coating.

In this study, we demonstrated the possibility of fabricating Ta-Hf-C coatings prepared by non-reactive DC magnetron sputtering. The sputtering process of Ta-Hf-C coating was optimized by adjusting substrate temperature and working pressure. The microstructure and mechanical properties of coatings were analyzed. The scratch behavior of Ta-Hf-C solid solution coating on hard brittle tungsten substrate and soft ductile 304 stainless steel substrate was investigated.

## 2. Experimental and Method

**Figure 1:**
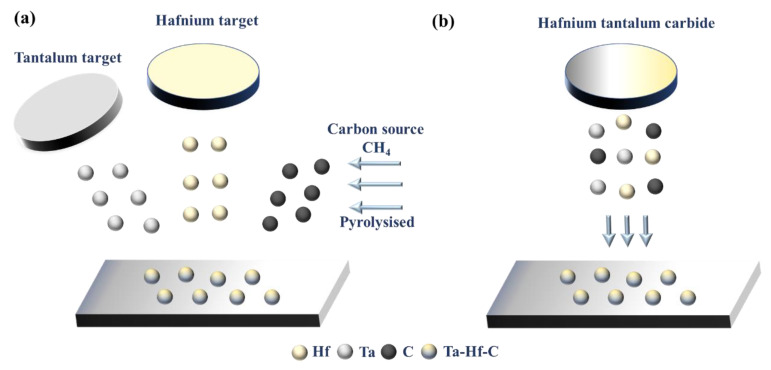
(**a**) Schematic diagram of reactive magnetron sputtered Ta-Hf-C solid solution coating, using pure hafnium and pure tantalum metal targets and methane as carbon source [9]; (**b**) schematic diagram of non-reactively magnetron sputtered Ta-Hf-C solid solution coating, using a hybrid target of HfC and TaC.

**Figure 2:**
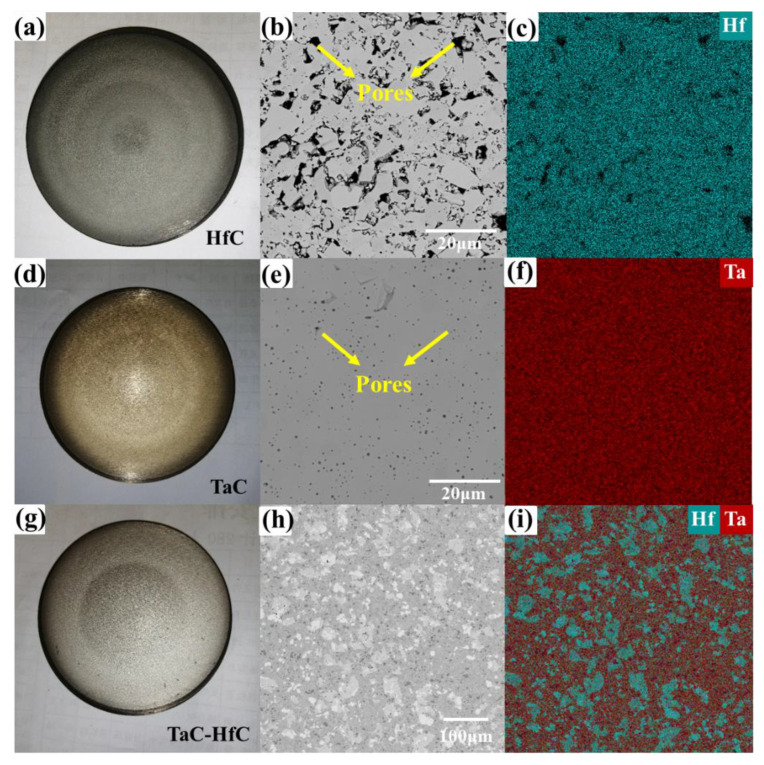
The optical images of the HfC (**a**), TaC (**d**), and TaC-HfC (**g**) target; the BSE images of surface morphology of HfC (**b**), TaC (**e**), and TaC-HfC (**h**); the corresponding EDS mapping results of HfC (**c**), TaC (**f**), and TaC-HfC (**i**).

The schematic diagram of reactive and non-reactive magnetron sputtering is shown in Figure 1. The key difference is that the carbon source of reactive magnetron sputtering is created by cracking methane molecules, while the carbon source of non-reactively magnetron sputtering comes from the target itself, and the non-reactive mode can be realized with only one target position, which reduces the cost and equipment requirements. In this paper, HfC, TaC, and Ta-Hf-C coatings were deposited by non-reactive DC magnetron sputtering using an JGP-450 system (Chinese Academy of Sciences) with Hf(Ta)C compound target and TaC-25 mol% HfC mixed target, respectively (purity: 99.9%, China Qi Jin New Materials Co. Ltd., Quanzhou, China). The morphology and composition of the targets are shown in Figure 2. HfC and TaC targets show a uniform composition structure with some acceptable sintering pores. The backscattered electron (BSE) and X-ray energy dispersive spectroscopy (EDS) results of TaC-HfC target show that the uniform mixing of Hf and Ta elements forms the composite with a small amount of mutual diffusion. The content of Hf is about 27 mol%, which is expected in the composite design. Obviously, there are no unexpected impurities in all targets.

The process optimization of Ta-Hf-C coating and the acquisition of HfC and TaC binary coatings all used tungsten as the substrate. Some Ta-Hf-C samples were deposited on 304 stainless steel substrates to study the effect of substrate characteristics on the scratch behavior. Before preparation, the substrate was cut into square plates with a dimension of 10 mm × 10 mm × 3 mm. The substrate surface was grinded with a 1000 mesh sandpaper and cleaned by ethanol solution. During the coating preparation, the background pressure in a deposition chamber was maintained at 6 × 10^−4^ Pa, and the target DC power was fixed at 80 W. The distance between target and substrates was kept at 10 cm. The deposition time of all coatings was 2 h and those process parameters are consistent in all deposition. Three working pressures of 0.4, 0.8, and 1.2 Pa were adopted by introducing argon gas (purity > 99.99%) with flow rates of 22.5 sccm, and the deposition temperature of the substrates was maintained at 400 °C. The influences of working pressure on the structure and mechanical properties of the Ta-Hf-C coating were discussed. Furthermore, the effect of deposition temperature (ambient temperature, 400 °C, 600 °C) on the coating was analyzed when the working pressure was 0.8 Pa.

The phase structures of the coatings were determined by X-ray diffractometry (XRD, Ultimate IV, RIGAKU, Tokyo, Japan) using CuKα radiation at a scanning rate of 4°/min. The morphologies and microstructures of the samples were obtained by a scanning electron microscope (SEM, TESCAN MIRA3, Oxford, UK) equipped with EDS operated at 15 kV voltage. Before SEM characterization, the cross-section of samples was grinded with 240, 400, 800, 1200 mesh silicon carbide papers in turn, and polished with diamond suspensions with a particle size of 0.5 μm. Nano-hardness and elastic modulus of coatings were characterized by nanoindentation (NHT2, Anton Paar TriTec, Peseux, Switzerland) using a Berkovich diamond. A load of 5 mN was utilized to avoid the effect of the substrate on hardness and elastic modulus of coatings. Five different positions were tested to obtain the average hardness of coatings through the standard deviation formula for each sample. The loading, holding, and unloading time were both 5 s. When the load was unloading to 10% of the maximum load, it should be maintained for 60 s to eliminate the influence of thermal drift. The hardness *H* and the elastic modulus *E* were estimated by using the Oliver–Pharr method [12]. The Poisson’s ratio of the coatings was considered to be 0.25 [13].

The micro-scratch performance of Ta-Hf-C solid solution coating was test by a Rockwell indenter with a radius of 100 μm (MST, Anton PaarTriTec, Peseux, Switzerland). A progressive loading mode with the load in the range of 10–5000 mN was applied. The loading rate and scratch rate are 5000/min and 500 μm/min, respectively. The acoustic emission acquisition rate was set at 30 Hz. The micrograph of scratch was obtained by an optical microscope (DM1750M, Leica, Wetzlar, Germany).

## 3. Results and Discussion

### 3.1. The Preparation Process Optimization of Ta-Hf-C Coating

**Figure 3:**
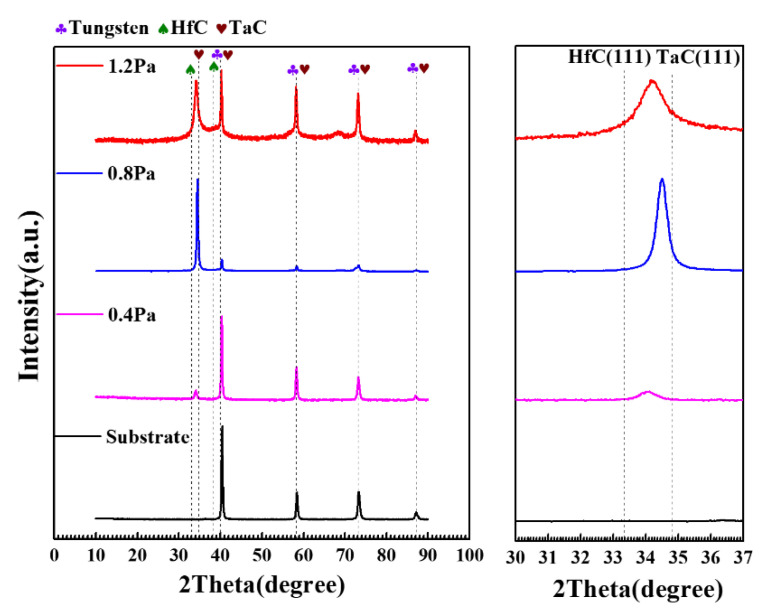
XRD patterns of Ta-Hf-C coatings under different working pressures.

**Figure 4:**
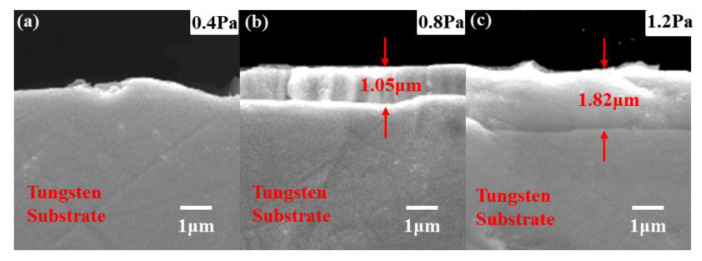
The SEM photographs of the Ta-Hf-C coating under different working pressures (**a**) 0.4Pa, (**b**) 0.8 Pa, and (**c**) 1.2 Pa.

**Figure 5:**
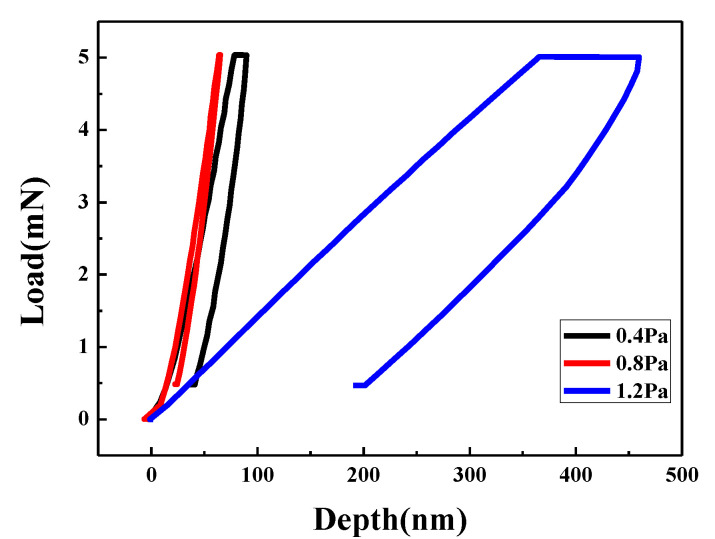
The typical load–displacement curves of Ta-Hf-C coatings under different working pressures.

Figure 3 shows the XRD results of Ta-Hf-C coating deposited at different working pressures. Based on the standard diffraction peaks of TaC (JCPDS No.35-0801) and HfC (JCPDS No.39-1491), the Ta-Hf-C ternary coatings with a face-centered-cubic phase are successfully obtained. The characteristic peak of (111) plane shifts significantly to the left compared with TaC, which is attributed to the fact that Hf atoms enter the TaC lattice to form a solid solution, and the lattice will expand because the radius of Hf (0.216 nm) atom is slightly larger than that of Ta atom (0.209 nm). The XRD patterns of the tungsten substrate show the characteristic peaks correspond to the cubic im3 m phases of tungsten (JCPDS-ICDD 04-0806). It is worth noting that the characteristic peaks of tungsten substrate are overlapped with some characteristic peaks of TaC and Ta-Hf-C, but it does not affect the judgment of the coating structure. With the increase of working pressure, the peak intensity of the (111) plane first increases and then decreases. When the working pressure is 1.2 Pa, the Ta-Hf-C coating has no preferred orientation and grows in the directions of (111), (200), (220), (222), and (400) crystal planes. Among them, the diffraction peak characteristics of (200), (220), (222), and (400) crystal planes are similar to those of tungsten in (110), (200), (211), and (220) crystal planes, respectively, so that the diffraction peak marked as W and TaC appears to be enhanced. The SEM cross-section morphology shows that the thickness of the Ta-Hf-C coating is difficult to detect when the working pressure is 0.4 Pa (Figure 4a), so the characteristic peak intensity of the coating is very weak. The coating thickness increases to 1.05 μm and 1.82 μm when the working pressures are 0.8 Pa and 1.2 Pa (Figure 4b,c). This is due to the fact that the lower working pressure reduces the number of incident atoms under the condition of constant sputtering power, while the sputtering yield of Ta and Hf is low owing to the large atomic number. Therefore, raising the working pressure leads to the increase of deposition rate. According to Scherrer relation [14], the increase in full width at half maximum (FWHM) at 1.2 Pa working pressure indicates that the grain size of the deposited coating further decreases. Figure 5 shows the typical nanoindentation load–displacement curves of Ta-Hf-C coatings under different working pressures. When the working pressures are 0.4 Pa and 0.8 Pa, the Ta-Hf-C coatings show the high hardness characteristic, reaching 27.47 ± 1.7 GPa and 37.8 ± 1.1 GPa, respectively. The difference may be due to the substrate effect caused by the coating thickness. However, when the working pressure is 1.2 Pa, the hardness of Ta-Hf-C coating reduces to 1.50 ± 0.2 GPa. There is a plastic deformation platform during the holding stage, which implies the metallicity of the coating. For non-reactively magnetron sputtering, the chemical composition of the coating may deviate from that of the target predictably, which is caused by any link in the deposition process, such as sputter angular distribution, gas scattering and etching effect, etc. The higher working pressure is considered to cause stronger scattering of the lighter C element in our study, resulting in the loss of carbon in the coating. This is consistent with the reports in literature [15,16]. The covalent bonding nature weakens with the decrease in carbon concentration, so the coating deposited at 1.2 Pa presents metallic characteristics.

Appropriate carbon loss has proven to be acceptable in previous reports, and the slightly substoichiometric HfC_x_ and TaC_x_ can achieve higher hardness and melting point (3928 °C for HfC_0.94_ and 3983 °C for TaC_0.88_) by adjusting the carbon vacancy concentration to cause the change of the electronic structure [17]. In general, the compounds composed of transition metals with high atomic number and light weight elements, such as C and N, are deposited by non-reactively sputtering, and the working pressure should be carefully controlled to avoid the excessive loss of light elements and maintain appropriate deposition efficiency.

**Figure 6:**
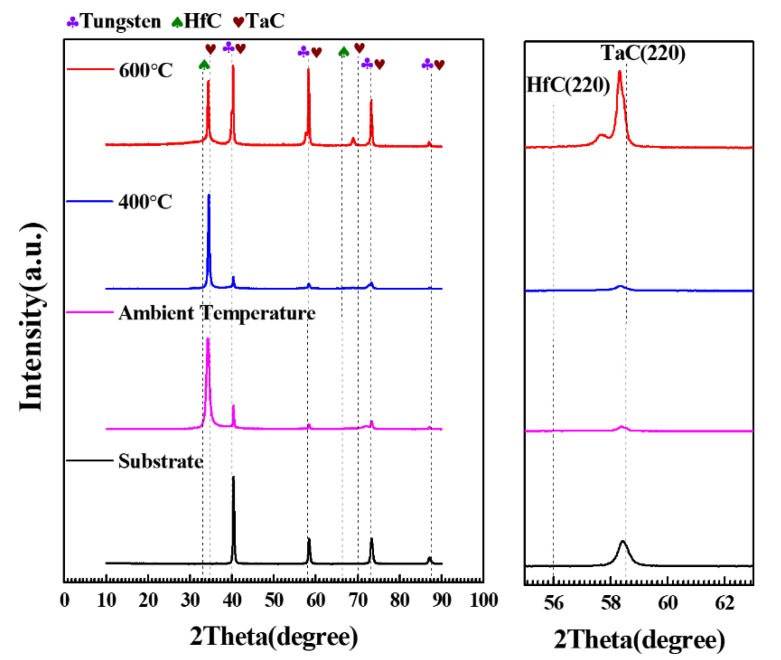
XRD patterns of Ta-Hf-C coatings under different deposition temperatures.

**Figure 7:**
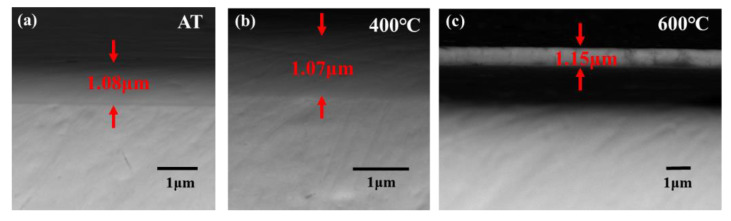
The SEM photographs of the Ta-Hf-C coatings under different deposition temperatures (**a**) Ambient temperature (**b**) 400 °C and (**c**) 600 °C.

**Figure 8:**
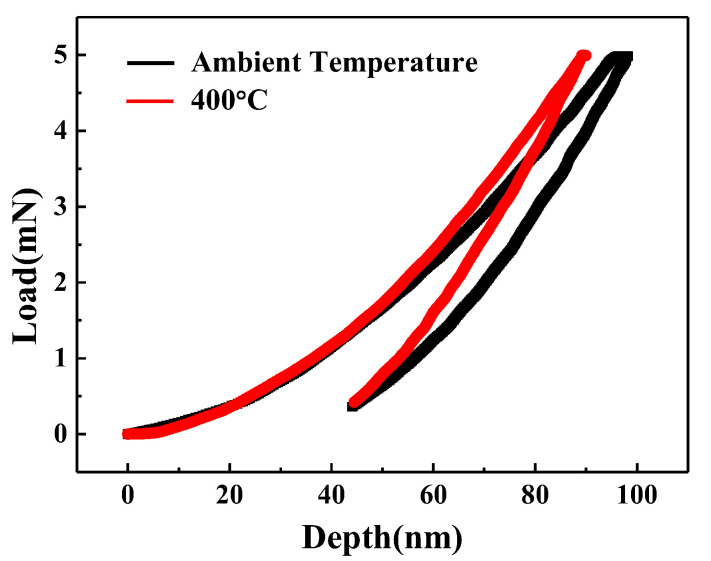
The typical load–displacement curves of Ta-Hf-C coatings under different deposition temperature.

Figure 6 shows the XRD patterns of Ta-Hf-C coatings deposited at different deposition temperatures. All coatings have an excellent crystallinity. The phase structure of the coating obtained at ambient temperature is similar to that at 400 °C, and the coating deposited at 600 °C has an additional growth on the (200), (220), and (222) crystal planes. The coating thickness is not significantly different when the deposition temperature is ambient temperature and 400 °C (Figure 7a,b), but the thickness increased slightly at 600 °C. With the increase of substrate temperature, the density of gases and ions near the substrate decreases. Therefore, Hf and Ta atoms sputtered from the target have little chance to be scattered, causing a small increase in the deposition rate. Unfortunately, excessive deposition temperature leads to cracking and spalling between substrate and coating due to thermal mismatch (Figure 7c). As shown in Figure 8, the coating deposited at 400 °C has a higher hardness compared with that at ambient temperature. This is attributed to the fact that the increase of substrate temperature increases the effective surface energy, which promotes the formation of energetically preferable structures so as to form closer stoichiometric compounds.

### 3.2. Microstructure and Mechanical Properties of HfC, TaC, and Ta-Hf-C Coating

**Figure 9:**
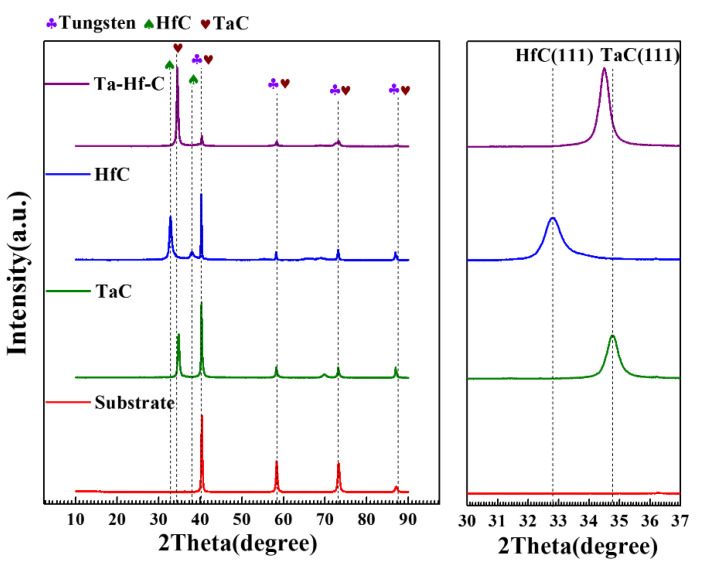
XRD patterns of TaC, HfC, Ta-Hf-C coatings, and the tungsten substrate. The inset shows the magnifications of the peaks in the 30–37° range.

Based on the results of process optimization, HfC, TaC, and Ta-Hf-C coatings were deposited at 0.8 Pa and 400 °C. Figure 9 shows the XRD patterns of three deposited coatings. The TaC (JCPDS No.35-0801) and HfC (JCPDS No.39-1491) coatings proved to be successfully obtained and no secondary phases were found. The XRD patterns of the Ta-Hf-C ternary coating show that when HfC is introduced into the sputtering target, the characteristic peaks HfC is not found in the coating, and the structure of the coating is still maintained in the face-centered-cubic TaC phase.

**Figure 10:**
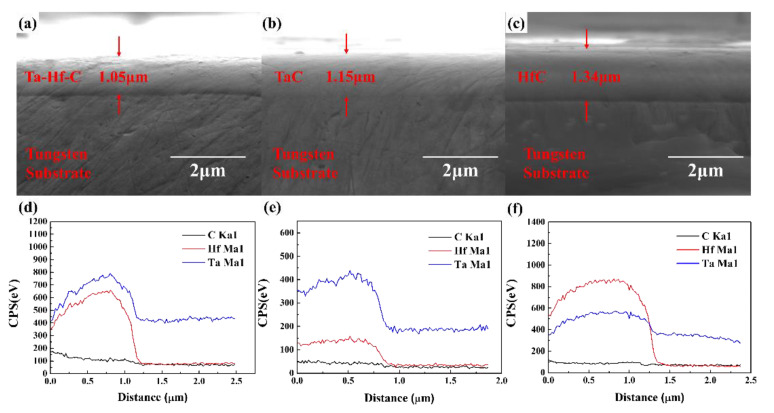
The SEM photographs of the Ta-Hf-C (**a**), TaC (**b**), and HfC (**c**) coating; The corresponding EDS line scanning results (**d**–**f**).

SEM photographs of the HfC, TaC, Ta-Hf-C coating, and the corresponding EDS line scanning results are shown in Figure 10. It can be seen that all the coatings are dense and tightly bonded with the tungsten substrate. The thicknesses of TaC, HfC, and Ta-Hf-C coatings after 2 h deposition are 1.15 µm, 1.34 µm, and 1.05 µm, respectively. The deposition rates of HfC and TaC coatings are 670 nm/h and 575 nm/h. This is attributed to the higher sputtering yield of Hf (0.37) compared to that of Ta (0.26) under similar Argon bombardment conditions [18]. The deposition rate of the Ta-Hf-C coating (525 nm/h) is slightly lower than that of TaC and HfC. This is contrary to the results of the previous confocal sputtering [8], where the deposition rate increases with increasing the sputtering ratio of Hf atom. The EDS line scanning results reflect that the ratio of Hf and Ta atoms in the Ta-Hf-C coating is about 15:21, which is higher than the target composition. This is also attributed to the higher sputtering yield of Hf. Furthermore, no uneven distribution of Hf and Ta is found, which means that the heterogeneous mixed target can achieve multi-component deposition with uniform composition through sputtering.

**Figure 11:**
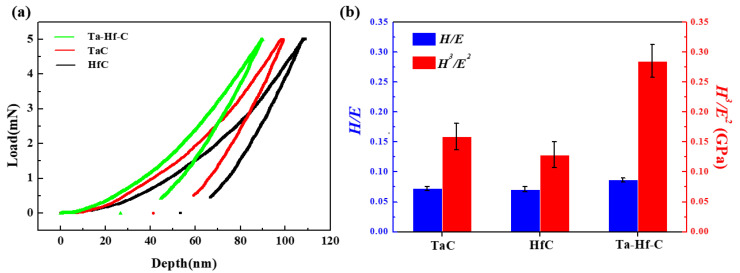
(**a**) The typical load–displacement curves of HfC, TaC, and Ta-Hf-C coatings; (**b**) *H*/*E* and *H*^3^/*E*^2^ plasticity index of HfC, TaC, and Ta-Hf-C coatings.

The typical load–displacement curves of the TaC, HfC, and Ta-Hf-C coatings are shown in Figure 11a. The hardness of TaC and HfC coatings are 30.2 ± 1.6 and 25.5 ± 1.2 GPa. The corresponding elastic modulus are 416.8 ± 5.9 GPa and 359.5 ± 10.8 GPa. Due to the solid solution strengthening effect, the local deformation caused by Ta/Hf atoms in Ta-Hf-C lattice makes the dislocation movement and the deformation more difficult, so that the hardness of the Ta-Hf-C coating is significantly higher than that of a single carbide. The hardness and elastic modulus of Ta-Hf-C solid solution coating both increase to 37.8 ± 1.1 GPa and 435.8 ± 13.8 GPa.

Plastic index *H*/*E* and plastic deformation resistance *H*^3^/*E*^2^ indicates the toughness and plastic deformation resistance of the coating. For wear-resistant coatings with protective effects, the higher the value of plastic index, the better [19]. The values of *H*/*E* of TaC, HfC, and Ta-Hf-C coatings are 0.072 ± 0.003, 0.071 ± 0.004, and 0.087 ± 0.003, respectively. The values of *H*^3^/*E*^2^ of TaC, HfC, and Ta-Hf-C coatings are 0.159 ± 0.022 GPa, 0.129 ± 0.021 GPa, and 0.285 ± 0.028 GPa, respectively, which are significantly higher than those of some traditional hard films [20,21]. The Ta-Hf-C solid solution coating has a higher plastic deformation resistance than the corresponding binary coating. This is confirmed by the ratio of the recovery depth to the maximum penetration depth of the nanoindentation. A recovery of around 70% is achieved for Ta-Hf-C solid solution coating. In contrast, the recovery of HfC and TaC coatings were about 50% and 58%, respectively. The introduction of Ta in the Ta-Hf-C would lead to the reduction of cohesive energy, which is the total energy required to break all bonds in a solid. The reduction of cohesive energy suggests the enhanced elastic properties [22].

### 3.3. Scratch Behavior of Ta-Hf-C Coating

**Figure 12:**
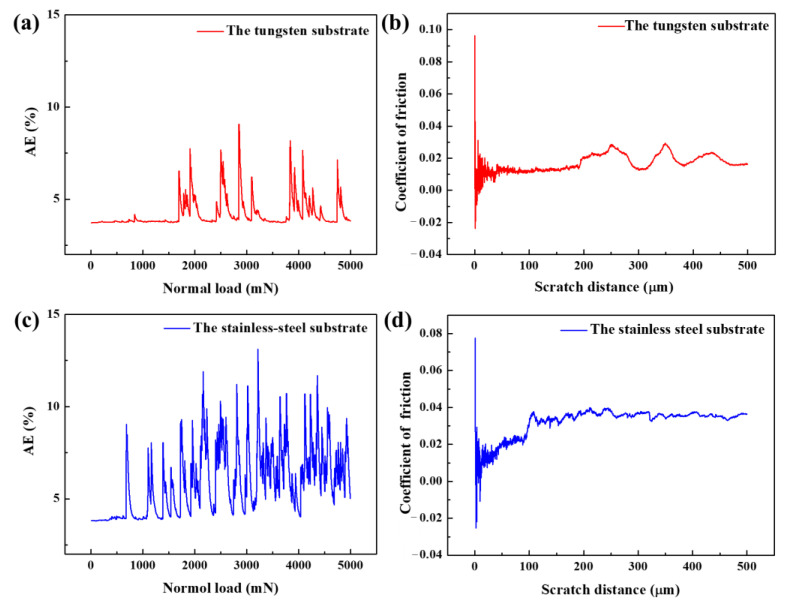
The acoustic emission signal and the friction curve of the Ta-Hf-C coating on tungsten substrate (**a**,**b**) and the stainless steel substrate (**c**,**d**).

**Figure 13:**
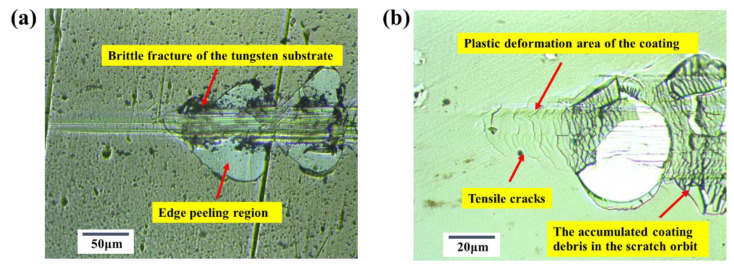
The scratched critical failure zone of Ta-Hf-C coating (**a**) on tungsten substrate (**b**) and the stainless steel substrate.

Figure 12a shows the acoustic emission signal of Ta-Hf-C coating on a tungsten substrate during the micro-scratch. It can be seen that the signal suddenly increases at 1700 mN, which implies the spalling failure of the coating occurs at this time. While the acoustic emission signal of Ta-Hf-C coating on stainless steel substrate (Figure 12c) sharply increases at 700 mN, the signal density and intensity are higher than that of the coating on tungsten substrate, which suggests that the critical load of coating failure is significantly lower than that of the coating on tungsten substrate. This confirms that the type of substrates are important aspects to determine the wear resistance and adhesion performance of brittle coatings. The scratch resistance properties are determined by the friction curves. The friction coefficient of Ta-Hf-C coating on a tungsten substrate achieves a very low level (~0.01) up to the scratch distance of about 200 μm (Figure 12b). The friction coefficient of Ta-Hf-C coating on a stainless steel substrate increases with increasing the scratch distance, and finally maintains at 0.035 (Figure 12d). The difference of the friction coefficient is that the stainless steel substrate is softer than the tungsten substrate, and more serious plastic deformation would occur between the indenter and the coating surface. The surface adhesion behavior combined with furrow effect leads to the rapid increase of the friction force, which is confirmed by the optical micrographs of the critical failure zone (Figure 13a,b). It is suggested that the plastic resistance of the coating depends not only on the material properties, but also on the mechanical properties of the corresponding substrate.

Compared with the stainless steel substrate, only a slight indentation groove formed along the scratch direction for the coating on tungsten substrate. Under the action of excessive stress, the brittle fracture of the tungsten substrate occurs first, leading to the emergence of indentation and the coating peeling of edge region. Soft and ductile substrate shows a stronger plastic deformation ability, the penetration depth of indenter, and the tensile force increases with the increase of the normal load. Only a plastic deformation area with tensile cracks appears in the critical failure zone. Next, the accumulated stress produces more cracks in the contour perpendicular to the scratch direction, the crushed coating is pushed into the scratch orbit, and the substrate is exposed. This leads to the drastic fluctuation of the friction coefficient of the coating. Therefore, the Ta-Hf-C coating with a high hardness and good plastic index is suitable for high hard and high toughness substrate, such as carbide tool, which can significantly improve the friction ability, corrosion resistance, and service life.

## 4. Conclusions

The TaC, HfC, and Hf-Ta-C single phase solid solution coatings are successfully prepared by non-reactively magnetron sputtering. The working pressure is carefully controlled to avoid the excessive loss of light elements and maintain appropriate deposition efficiency. Proper deposition temperature can improve the mechanical properties of the coating. The optimum preparation process of working pressure and deposition temperature are 0.8 Pa and 400 °C, respectively. Due to the solid solution strengthening effect, the local deformation caused by Ta/Hf atoms in Ta-Hf-C lattice makes the dislocation movement and the deformation more difficult, so that the hardness of the Ta-Hf-C coating is significantly higher than that of a single carbide. Compared with the pure HfC binary coating, the hardness, elastic modulus, and plastic deformation resistance (*H*^3^/*E*^2^) of ternary Ta-Hf-C coating are significantly increased by about 48%, 21%, and 220%, respectively. The micro-scratch critical failure loads of Ta-Hf-C coating on tungsten substrate and the stainless steel substrate are about 1700 mN and 700 mN, respectively. The friction coefficient of the Ta-Hf-C coating on tungsten substrate (0.01) is lower than that on the stainless steel substrate (0.035). The plastic deformation resistance of Ta-Hf-C coating depends on the mechanical properties of the substrate.

## Data Availability

The data presented in this study are available on request from the corresponding author.
